# Exploring the impact of terminology differences in blood and organ donor decision making

**DOI:** 10.1371/journal.pone.0227536

**Published:** 2020-01-09

**Authors:** Stephen Whyte, Ho Fai Chan, Karin Hammarberg, Benno Torgler

**Affiliations:** 1 School of Economics and Finance, Queensland University of Technology, Gardens Point, Brisbane, QLD, Australia; 2 Centre for Behavioural Economics, Society and Technology, Queensland University of Technology, Brisbane, QLD, Australia; 3 Centre in Regenerative Medicine, Institute of Health and Biomedical Innovation, Queensland University of Technology, Kelvin Grove, QLD, Australia; 4 School of Public Health and Preventive Medicine, Monash University, Melbourne, Victoria, Australia; 5 CREMA—Center for Research in Economics, Management and the Arts, Zurich Switzerland; Middlesex University, UNITED KINGDOM

## Abstract

Because the global shortage of blood and organ donors across all medical markets is a serious concern for health care provision, we aim in this study to better understand decisions (not) to participate in these two forms of medical donation, which can save or prolong another’s life. Using unique responses from over 1,000 online survey respondents, we compare the reasons given for the donation decision given by blood and/or registered organ donors versus non-donors. To do so, we categorize responses based on five dimensions of language choice: egocentric (referring to self), social, moral, positively emotional, and negatively emotional. Our results reveal statistically significant differences between blood donors and non-donors in the use of all five categories. With respect to organ donation, we find statistically significant differences between donors and non-donors in the use of social, moral and positive emotional terms but not in the use of egocentric or negatively emotional justifications. Such results suggest that the ‘gift of life’ terminology used universally to market to potential blood and organ donors may only be relevant in the blood donation market and unlikely to incentivize or change organ donation behaviour.

## Introduction

The marketing and promotion of medical donation often frames the act as giving ‘the gift of life’, indicating the potential for social capital accrual by the gift giver [1–4]. A form of reciprocal altruism, a behaviour that temporarily reduces fitness, and can have an important evolutionary, cultural, and emotional in-group effect[5,6]. Yet, although not-for-profit, public, and private organisations use this phrase frequently and consistently across all forms of medical donation, blood and organ donation (permission for donation, after death) are very different in their medical, psychological, and socio-cultural implications. In particular, the two differ in how individuals give consent and supply their donation, the level of time and physical investment required for donation, the timing of when a donation can be given (or taken), and, most significantly, their *ex-ante* and *ex-post* physical and psychological costs. In fact, the only immediately quantifiable similarity between blood and organ donation is that they continue to be under-supplied in developed economies[7–10]. A gamut of donation literature indicates that even though organizations call continually for such an altruistic and non-reciprocal medical gesture–one that has significant benefits for ‘free-riders’ who ignore such donor appeals—most people do not and never will donate[8,11].

Not surprisingly, then, long before (and ever since) Titmuss’s (1970) seminal work on blood donation markets *The Gift Relationship*, researchers have sought to study and understand the interplay of social, cultural, economic, and ethical factors in the decision to become a donor. Both currently and historically, commercial blood and organ donation organizations have been based primarily on either an altruistic or financially remunerated model whose varying incentive structures attract individuals with different underlying motivations for participation[11]. Remuneration incentives have been shown to be important early motivators for donation[12,13]. A gamut of literature has also shown donors report altruistic motivations as their primary driver for donating[14–17]. Indeed studies have shown blood donors to be higher in primary prosocial characteristics like altruism, empathy and social responsibility, which are commonly thought to be the key drivers motivating donation[18]. However, it is unclear how the way the term altruism is perceived by the individual in communications from health organisations and the subjective nature of altruism itself. As such, there is a significant debate on whether altruism is the major motive underlying donation[15]. This is because no single incentive or factor has been identified that all segments of donors and non-donors report positive attitudes and behaviours towards[19]. This may be because participants’ preferences are sensitive to framing[20,21], and that prosocial behaviour may in instead be driven by a more generalized preference for morality and “*doing the right thing*”[22]. Hence, the factors that affect (negative) perceptions of and non-participation in donation markets, for the majority of the population who have never considered or had the opportunity to donate, remain unclear. Asking individuals why they do *not* donate blood or register as an organ donor may thus provide more valuable insights into how to increase donor numbers than simply asking self-selected donors to rationalize (*ex-post*) why they *do* donate. Currently, in Australia organ donation registration is as costless as ticking a box during driver’s license renewal or completing tax return. Consent to be an organ and tissue donor are recorded on the Australia Organ Donor Register, which can be altered at any time at will, including the decision not to donate. Blood donation, on the other hand, requires individuals to attend a blood donation facility for an extended period of time to donate. For the purposes of this study, we explore the self-reported differences between those who have and have not, physically donated blood, or officially registered as an organ donor.

Because familiar or standardized terminology is such an important part of health organizations’ communications with potential and existing donors, this study explores key differences in the language that donors and non-donors associated with blood and organ donation. Understanding key egocentric, social, moral, and emotional factors that impact and drive individual decisions to (not) participate in medical donation can provide vital insights into how to foster, support, and motivate increased donation and altruistic or prosocial behaviour across blood and organ donation markets both now and in the future.

## Materials and methods

### Data capture

Anonymous data were collected using the Queensland University of Technology (QUT) KeySurvey software between July 13 and September 7, 2017 using an online survey advertised to potential participants primarily via social media. Several Australian donation organizations, including Zaidee’s Rainbow Foundation, Kidney Health Australia, and Transplant Australia, assisted in promoting the study to their social media platform users to ensure part of the sample included people with a history of donation. As with all social media advertising, cross-promotion of the study to and by other organisations and websites was not within the control of the researchers (e.g., referral links via social media or other platforms). It is therefore not possible to accurately account for the conduit to which the participants self-select into the survey. Because donors are distinctly underrepresented in the general population, the researchers specifically targeted donation websites to ensure a large donor sample population and statistical power in our comparative analysis. Targeting donation organisations and utilising their social media platforms also provided the researchers the opportunity to sample, arguably, “informed” non-donors. Conservatively, one could conclude that these individuals may be more exposed to our partner donation organizations’ social network or are more connected to people who are, and thus, may have higher likelihood of becoming a future donor. Nevertheless, such individuals are in part representative of the engaged non-donor population that blood and organ donation charity organisations, the healthcare sector, and policymakers alike, specifically wish to engage and influence the behaviour of.

The study was conducted in accordance with the protocol approved by the Human Research Ethics Committee at the Queensland University of Technology (approval review no. 1700000421). All participants provided informed written consent upon completion of the survey.

Survey participants, who were incentivized to participate through five prize draws (lotteries) of AUD $100 cash, were first asked to provide socio-demographic information (e.g., year of birth, height, weight, educational level, marital status, sexuality, and income) and then asked if they had ever donated blood or were currently a registered organ donor. Depending on their responses, they were then asked to provide a short written response (approximately two sentences) in their own words on the ‘feelings or reasons’ underlying why they had or had not chosen to be a blood donor or register as an organ donor.

Some examples of responses provided:

**Respondent X—***It's something I wouldn't mind doing someday*, *but it's not something that's on my mind often and I haven't found the right opportunity at the right time yet (e*.*g*., *a blood bank drive or a donation van that I have time to visit when I see it)*

**Respondent Y**–*It’s really feels fully satisfied after donating blood*. *I donated more than 5 times*. *Seeing the recipient relaxed face I was motivated more to donate again*. *Donating blood to someone's need will certainly saves one’s life*.

***Respondent Z—**It freaks me out (surgery, my body being operated on, my body being exposed/naked), and I have conflicted feelings about the ethics of prolonging life due to organ failure and where we're going as a species in our need to continue prolonging human life and its impact on the Earth and our ecosystems. So I'd rather abstain from making any decision and leaving the decision up to my partner/family when I die. I am fine with my partner doing whatever he feels will help his grieving process. But I'd like to live with the knowledge that my body will not be operated on upon death*.

### Participant descriptive statistics

**Fig 1:**
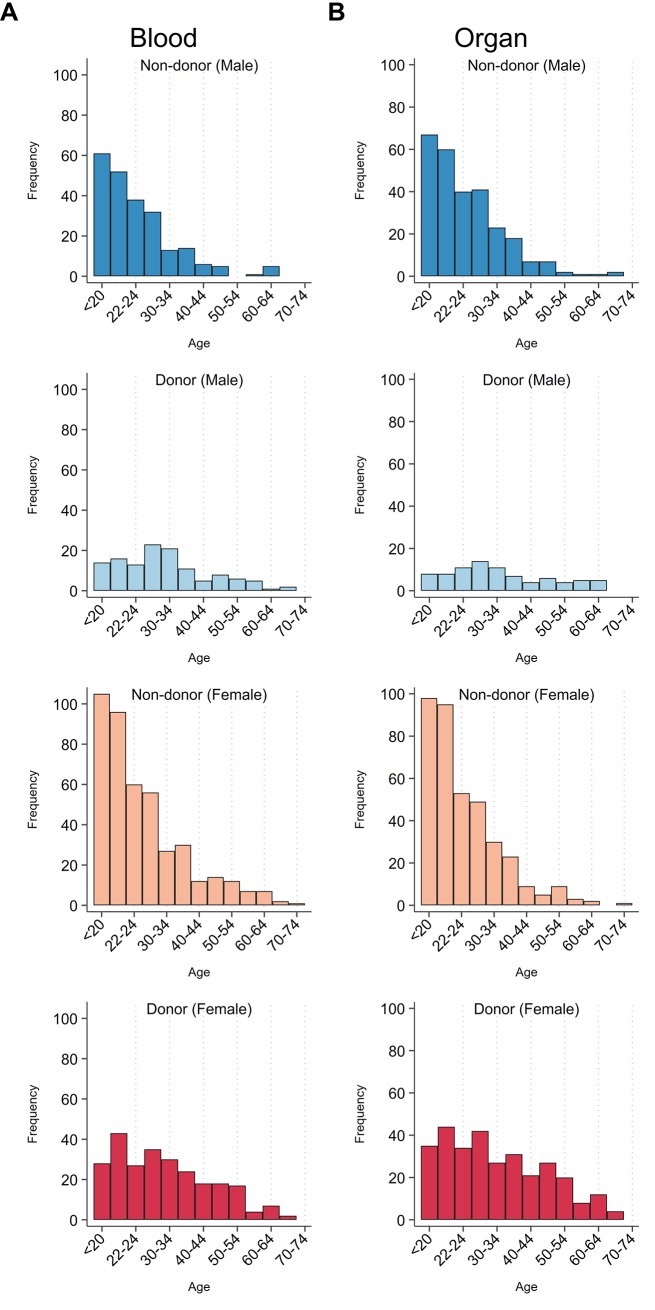
Age and sex distribution, by donation history. Blood donation (A): Sample size of male donor = 125; male non-donor = 227; female donor = 254; female non-donor = 429. Organ donation (B): Sample size of male donor = 83; male non-donor = 269; female donor = 306; female non-donor = 377.

Of the 1,035 respondents, 683 (66%) were female and 352 (34%) were male, with an age range from 18 to 70 years (male: *M* = 27.43, *SD* = 10.67; female: *M* = 28.85, *SD* = 11.80). Fig 1 reports the frequency distribution of participants’ age by their donation history in both forms of donation, and differentiated by sex. Blood (organ) donors make up 36.62% (37.58%) of the entire sample. 44.25% (*N* = 458) of participants reported having never donated blood nor currently being registered as an organ donor. 198 (19.13%), 188 (18.16%), and 191 (18.45%) participants are blood-only, organ-only, and both blood and organ donor, respectively. Although the proportions of female and male blood donors are similar (*P* = 0.596, two-tailed test), significantly more women than men were registered as organ donors (*P* < 0.001, two-tailed test). The average height and weight of male participants is 177.3 cm (*SD =* 9.11) and 76.56 kg (*SD* = 15.16); and 165.18 cm (*SD =* 7.32) and 65.06 kg (*SD =* 14.64) of female participants. Most respondents self-identified as heterosexual (*N* = 832, 80.39%), with the remainder identifying as bisexual or pansexual (*N* = 125, 12%), gay or lesbian (*N* = 34, 3.3%), asexual (*N* = 7, 0.7%), or other (*N* = 37, 3.6%). More than half the respondents (*N* = 603, 58.3%) reported being single; the remainder were married (*N* = 214, 20.7%) or in a de facto relationship (*N* = 137, 13.2%). One in five participants had children at the time of the survey (*N* = 213, 20.6%). Most respondents (*N* = 666, 64.4%) had completed or were in the process of completing some form of post-secondary study (diploma, technical college, undergraduate, postgraduate or PhD), 34.11% (*N* = 353) had completed high school (Grade 12) but 1.4% *N* = 15) had not. Most earned AUD$40,000 or less per annum (*N* = 775, 74.9%), with 18% earning between AUD$40,001 and $200,000 AUD; and 5.3% (55) chose not to provide income information. Lastly, 41.1% of the respondents (*N* = 425) reported being atheists, while 29.5% (*N* = 305) self-identified as Christians. Other religious affiliations in the sample included “Other” (*N* = 166, 16.04%), Buddhism (*N* = 54, 5.22%), Islam (*N* = 49, 4.73%), Hinduism (*N* = 32, 3.09%) and Judaism (*N* = 4, 0.39%).

In S1 to S3 Tables (see supplementary material), we present logistic and multinomial regression exploring factors that predict blood and organ donation, differentiated by sex and type of donation, respectively. In line with previous research, our sample population showed age to be positive correlate with participants’ likelihood of being a blood donor[23,24], as was being male for organ donation[25]. Further, our odds-ratio analysis aligns with the bulk of the literature showing religious beliefs and cultural influences appear to have a distinctly negative impact on both blood donation rates and willingness to sign up as (cadaveric) organ donor[26–29].

### Variable measures

To quantitatively analyse the responses of donors and non-donors, which are qualitative in nature, we utilized the Linguistic Inquiry and Word Count (LIWC) dictionary and the Moral Foundations dictionary (MF) to construct our dependent variables[30–32]. Specifically, we selected four categories from the LIWC dictionary to explore the egocentric (‘I’ category, with first-person singular pronouns), social references (‘social’ category,), and emotional (‘positive emotion’ and ‘negative emotion’ categories) aspects of the responses. For the MF dictionary, we used the general category to examine the presence of moral sentiment as well as positive aspects of the *Care* and the *Fairness* foundations which correspond to “ethic of care” and “ethic of justice”, respectively. We choose these categories because research has repeatedly shown that donors often cite pro-social motivations and reasons when describing their decision to donate, while non-donors often allude to negative emotions associated with the donation process[18,33–34]. We thus measure the share of individual words (or word-stems) from each category used (i.e., the total number of times any word (or word-stem) in the category appear in the response text, calculated as a percentage of the total word count of the response text). The LIWC egocentric category contains 14 first-person singular pronouns, the social category contains 457 words, and the positive and negative emotions categories contain 500 and 406 words, respectively. The “general morality” category of the MF dictionary encompasses a total of 41 words, whereas the positive aspects of *care* and *fairness* foundations each have 16 and 26 words.

## Results

**Fig 2:**
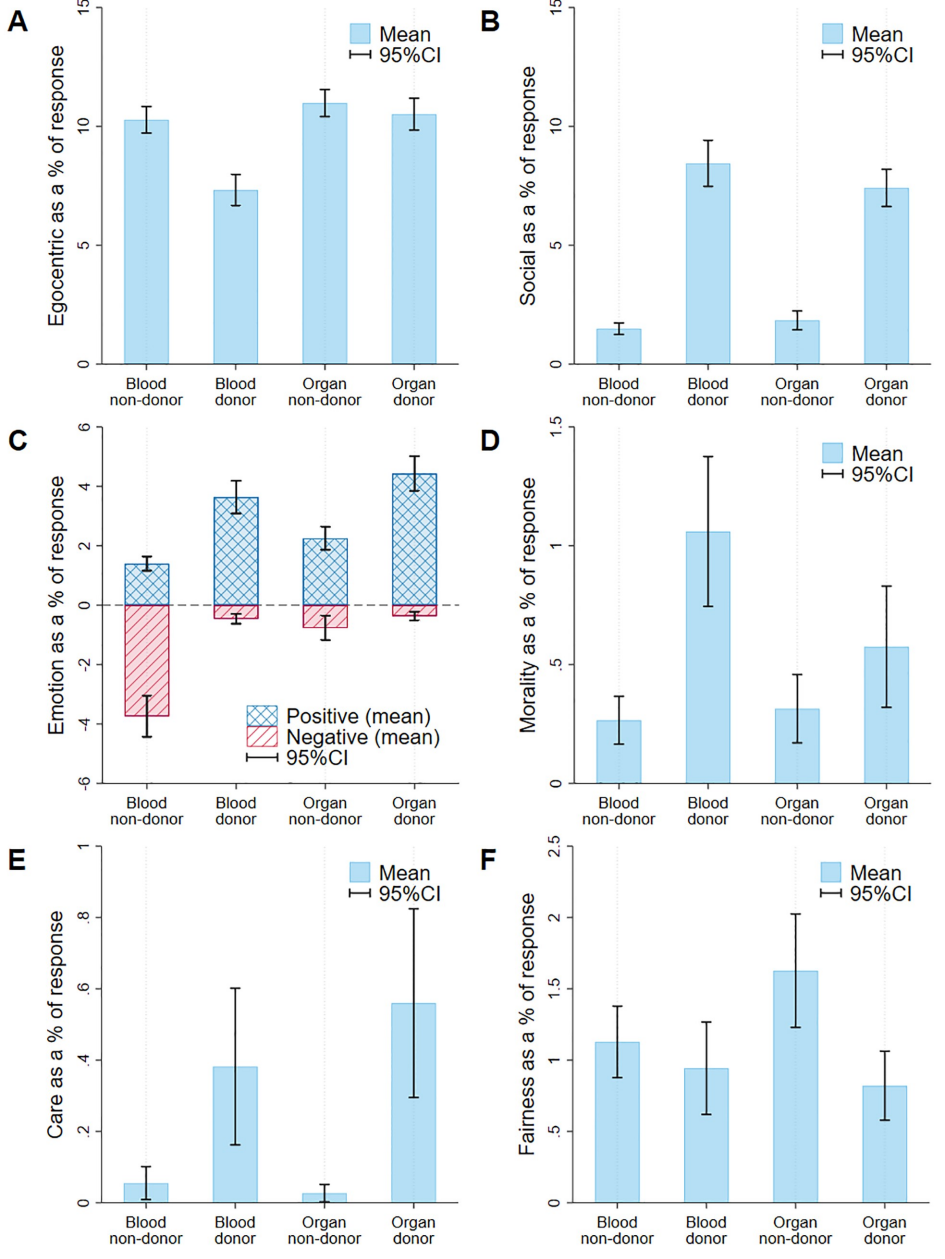
Percentage of words used in responses.

In Fig 2A–2F, we graphically depict the mean and 95% confidence interval for each category of donor and non-donor across the four LIWC and MFD categories of interest (i.e., egocentric, social, moral, positive emotion, and negative emotion). To control for the statistically larger keyword percentages generated by shorter written responses, we as a percentage of the total number of individual words provided. We find that whereas the use of first-person egocentric terminology accounts for approximately 7% of responses among blood donors, for blood non-donors, organ donors, and non-organ donors, it falls into the 10%-11% range (Fig 2A). In contrast, less than 2% of blood and organ non-donors’ responses contain words from the LIWC social category, although on average they account for between 7.5% and 8.5% of total responses from the blood and organ donors, respectively (Fig 2B).

We graph a visually continuous scale of negatively and positively emotional responses but do not combine negative and positive variables so as not to overweight or bias disproportionate responses for a particular group (Fig 2C). The count for negatively emotional responses is similar (between 0% and about 1%) for all groups except non-blood donors, for whom it is close to 4%. On the other hand, whereas non-donors of both categories used an average of 1% to 2.5% positively emotional responses, the usage among donors of both categories is almost twice as high (between 3.5% and 5%).

Finally, in Fig 2D to 2F we graphically depict the use of moral terminology by participants with both categories of donors (compared to non-donors) showing statistically significantly higher use of terms relating to general morality and *care* foundation in the *ex-post* rationalization of their donation decisions. Blood donors, in particular, using close to four times more morality-based terminology when compared to non-donors. In contrast, both blood and organ non-donors report higher usage of words from the *fairness* foundation than their donor counterparts, with organ non-donors doubling the usage compared to organ donors.

Hence overall, as demonstrated by the Wilcoxon rank-sum and *t*-test comparisons performed for all groups, the only word categories that show no statistically significant differences between donors and non-donors are the egocentric and negative emotion words used by organ donors versus organ non-donors and fairness foundation words used by blood donors versus blood non-donors (Table 1). In addition, we check if blood donor or non-donor responses differ by whether they are also organ donor or non-donor, and *vice versa*. We find no statistically significant differences in most categories examined (see S4 and S5 Tables).

**Table 1:** Difference in responses between donors and non-donors in blood and organ donation.

Donation	Category	N_donor_	N_non-donor_	Z-stat.	*p*-value	Mean diff.	Cohen’s d [95%CI]
Blood	*Egocentric*	379	656	6.42	< 0.001	-2.96***	-0.42 [-0.55,-0.29]
Blood	*Social*	379	656	-17.1	< 0.001	6.95***	1.1 [0.96,1.24]
Blood	*Pos emo*	379	656	-7.98	< 0.001	2.24***	0.55 [0.42,0.67]
Blood	*Neg emo*	379	656	8.44	< 0.001	-3.28***	-0.45 [-0.58,-0.32]
Blood	*Morality*	379	656	-5.8	< 0.001	0.77***	0.36 [0.23,0.49]
Blood	*Care*	379	656	-3.73	< 0.001	0.33**	0.23 [0.36,0.11]
Blood	*Fairness*	379	656	0.98	0.327	-0.190	-0.06 [0.07,-0.18]
Organ	*Egocentric*	389	646	1.05	0.292	-0.470	-0.07 [-0.19,0.06]
Organ	*Social*	389	646	-16.65	< 0.001	5.57***	0.89 [0.76,1.02]
Organ	*Pos emo*	389	646	-8.94	< 0.001	2.18***	0.41 [0.28,0.53]
Organ	*Neg emo*	389	646	-0.64	0.521	-0.40†	-0.09 [-0.22,0.03]
Organ	*Morality*	389	646	-3.59	< 0.001	0.30*	0.15 [0.02,0.27]
Organ	*Care*	389	646	-6.53	< 0.001	0.53***	0.32 [0.45,0.20]
Organ	*Fairness*	389	646	1.74	0.082	-0.81***	-0.19 [-0.06,-0.31]

Sample size: Blood donors (N = 379), non-donors (N = 656), organ donors (N = 389) and non-donors (N = 646). *z*-statistics and *p*-values are from the Wilcoxon rank-sum test (two-tailed). Mean comparison (in percentage points) using *t*-test (two-tailed).

†, *, **, and *** represent statistical significance at the 10%, 5%, 1%, and 0.1% levels, respectively.

### Multivariate analysis

Table 2 shows the results of our multivariate ordinary least squares (OLS) regression analysis of the differences in LIWC and MFD category responses using the respective word counts from each as the dependent variable. As previously explained, this variable represents seven categories of egocentric, social, moral (general, *care* and *fair*), and positively and negatively emotional while controlling for the number of words provided in each response. Each model also includes multiple independent control variables (including age, sex, height, income, educational level, religious affiliation, parental status, and sexuality).

**Table 2:** Factors associated with the use of LIWC categories among donors and non-donors.

*Dep*. *Var*.	*Egocentric*		*Social*		*Positive Emotion*		*Negative Emotion*	
Sample	Blood	Organ	Blood	Organ	Blood	Organ	Blood	Organ
*Indep*. *Var*.	(1)	(2)	(3)	(4)	(5)	(6)	(7)	(8)
Donor	-3.03***	-0.36	6.79***	5.45***	2.27***	2.16***	-3.38***	-0.11
	(-6.54)	(-0.69)	(13.59)	(11.41)	(7.23)	(5.31)	(-8.50)	(-0.68)
Male	-0.07	0.82	-0.01	-0.46	0.17	-0.31	-0.17	1.04
	(-0.11)	(1.10)	(-0.02)	(-0.66)	(0.50)	(-0.80)	(-0.25)	(1.41)
Age	-0.05†	-0.01	0.02	-0.05	-0.05***	-0.01	-0.03	-0.01
	(-1.66)	(-0.29)	(0.46)	(-1.60)	(-3.32)	(-0.33)	(-1.38)	(-1.22)
Height (cm)	-0.04	-0.02	-0.01	0.00	-0.00	-0.02	0.02	-0.06
	(-1.30)	(-0.43)	(-0.22)	(0.08)	(-0.09)	(-0.68)	(0.50)	(-1.64)
Weight (kg)	-0.02	-0.01	0.01	0.01	0.01	-0.00	-0.01	0.00
	(-1.38)	(-0.59)	(0.90)	(1.02)	(0.68)	(-0.09)	(-0.83)	(0.02)
*ln*(Income)	-1.02*	0.23	0.15	0.11	-0.11	-0.34	-0.47	-0.11
	(-2.54)	(0.50)	(0.36)	(0.28)	(-0.41)	(-1.17)	(-1.19)	(-0.67)
*Education*								
High School	0.48	0.08	0.05	-0.80†	-0.32	0.61	-1.70**	0.04
	(0.87)	(0.14)	(0.13)	(-1.70)	(-1.11)	(1.50)	(-2.97)	(0.09)
Post-Graduate	1.06†	-0.78	-0.06	0.38	0.35	0.22	1.50†	0.15
	(1.71)	(-1.15)	(-0.09)	(0.58)	(0.92)	(0.45)	(1.75)	(0.56)
Single	-0.49	-0.91	-0.74	-0.25	0.09	-0.47	0.39	-0.05
	(-0.86)	(-1.60)	(-1.39)	(-0.52)	(0.28)	(-1.17)	(0.70)	(-0.25)
*Religion*								
Buddhism	-0.82	-1.38	0.39	1.10	0.61	0.41	0.47	1.95
	(-0.73)	(-1.11)	(0.39)	(0.94)	(0.72)	(0.45)	(0.35)	(1.62)
Christianity	-0.13	-0.15	0.41	1.14*	0.25	0.84*	1.20†	-0.09
	(-0.24)	(-0.27)	(0.91)	(2.33)	(0.74)	(2.12)	(1.89)	(-0.39)
Hinduism	0.94	-0.68	0.38	0.66	1.96	1.60	-0.92	-0.69
	(0.76)	(-0.48)	(0.35)	(0.65)	(1.53)	(1.52)	(-0.92)	(-1.43)
Islam	-1.08	-0.16	0.29	1.08	-0.70	1.45	-0.31	-0.51
	(-1.02)	(-0.11)	(0.23)	(1.07)	(-1.26)	(1.46)	(-0.39)	(-0.74)
Judaism	-2.34	2.89	-1.20†	-1.04	-1.96**	-1.67	1.80	-0.60*
	(-1.14)	(0.97)	(-1.73)	(-0.26)	(-2.85)	(-0.82)	(1.39)	(-2.16)
Other	-0.26	0.69	0.46	0.49	-0.54†	1.05†	0.35	-0.02
	(-0.39)	(1.00)	(0.73)	(0.88)	(-1.72)	(1.73)	(0.52)	(-0.04)
Childless	-1.22	0.38	-0.30	-1.28	-0.52	-0.43	0.73	-0.47
	(-1.64)	(0.41)	(-0.34)	(-1.53)	(-1.06)	(-0.74)	(1.28)	(-1.13)
Heterosexual	0.18	0.39	-0.41	-0.35	0.33	0.37	-0.40	-0.99
	(0.29)	(0.64)	(-0.82)	(-0.60)	(1.11)	(0.93)	(-0.59)	(-1.47)
N	977	977	977	977	977	977	977	977
*R*^*2*^	0.08	0.01	0.24	0.17	0.09	0.05	0.07	0.04
Prob. > *F*	0.000	0.748	0.000	0.000	0.000	0.000	0.000	0.313

*Notes*: Coefficient estimates obtained from ordinary least squares (OLS) regressions. *t*-statistics are given in parentheses, and standard errors are robust to heteroskedasticity.

The references for educational level and religion are college degree and atheism, respectively.

†, ***, **, and * denote significance at the 0.1%, 1%, 5%, and 10% levels, respectively.

According to models 1 and 2, which compare blood and organ donation responses that include egocentric category terms, blood donors use fewer personal reference words than non-donors when justifying their *ex-post* decision to donate. This lower use of egocentric terms is also associated with older age, being taller, and having a higher income. Models 3 and 4 then compare the responses that include social category words, showing that both blood and organ donors (versus non-donors) are more likely to include social terminology in their donation justifications. Christians are also more likely than atheists to use social category terms.

The results for models 5 and 6, which compare responses that include positive emotions category terms, are similar to those for the social category: blood and organ donors (relative to non-donors) are more likely to use positively emotional words when justifying their donor choice, a usage that is also positively associated with age. Similarly, the model 7 and 8 results for responses that include negatively emotional words show that blood donors use fewer of these latter in their responses than non-donors. Nonetheless, mirroring the findings for egocentric responses, no statistically significant differences in the use of these latter are observable between organ donors and non-donors.

In Table 3 we compare responses that include moral category terms. In models 9 and 10 we see only statistically significant results for blood donors (compared to non-donors) who state more moral terms in their explanations. In models 11 and 12 we see organ donors (compared to non-donors) use more words related to care foundations. Interestingly, in models 13 and 14 we see that those who have chosen not to be organ donors (compared to donors) state more fairness foundation words.

**Table 3:** Factors associated with the use of MF categories among donors and non-donors.

*Dep*. *Var*.	*Morality*		*Care Foundation*		*Fairness Foundation*	
Sample	Blood	Organ	Blood	Organ	Blood	Organ
*Indep*. *Var*.	(9)	(10)	(11)	(12)	(13)	(14)
Donor	0.75***	0.12	0.25*	0.51***	-0.18	-1.04***
	(4.35)	(0.88)	(2.33)	(4.19)	(-0.92)	(-3.46)
Male	0.09	0.03	0.03	0.09	0.67*	0.05
	(0.52)	(0.22)	(0.21)	(0.83)	(2.04)	(0.13)
Age	-0.00	0.00	-0.00	-0.01	-0.02	0.03
	(-0.51)	(0.57)	(-0.42)	(-0.71)	(-1.31)	(1.39)
Height (cm)	-0.01	-0.00	-0.01	-0.01	-0.02	-0.02
	(-0.77)	(-0.50)	(-0.77)	(-1.27)	(-1.06)	(-1.15)
Weight (kg)	0.00	0.00	0.00	-0.00	0.01	0.01
	(0.44)	(0.54)	(0.55)	(-0.44)	(0.86)	(0.92)
*ln*(Income)	0.07	0.03	0.06	0.07	0.23	0.20
	(0.44)	(0.22)	(1.10)	(0.67)	(0.95)	(1.07)
*Education*						
High School	-0.02	0.15	-0.06	-0.01	0.30	0.25
	(-0.16)	(1.21)	(-0.60)	(-0.10)	(1.20)	(0.74)
Post-Graduate	0.30	-0.11	-0.13	0.09	-0.08	-0.49
	(1.51)	(-0.60)	(-0.79)	(0.46)	(-0.29)	(-1.22)
Single	-0.04	-0.11	0.18*	-0.01	-0.33	0.00
	(-0.28)	(-0.71)	(1.98)	(-0.07)	(-1.20)	(0.00)
*Religion*						
Buddhism	0.39	0.04	0.47	-0.05	0.19	-0.91*
	(0.80)	(0.22)	(1.15)	(-0.24)	(0.32)	(-2.20)
Christianity	0.17	0.15	0.01	0.15	-0.38	-0.63*
	(0.88)	(1.40)	(0.09)	(0.94)	(-1.57)	(-2.49)
Hinduism	-0.16	0.88†	1.41†	-0.06	-1.10***	-0.18
	(-0.42)	(1.65)	(1.65)	(-0.52)	(-3.73)	(-0.27)
Islam	-0.59**	0.14	0.34	-0.17	-0.65	2.54
	(-3.28)	(0.70)	(0.86)	(-1.59)	(-1.53)	(1.30)
Judaism	-0.37	-0.38*	-0.04	-0.50*	-1.22***	1.13
	(-1.46)	(-2.16)	(-0.34)	(-2.09)	(-3.91)	(0.94)
Other	-0.05	0.39	-0.08†	-0.12†	0.08	-0.38
	(-0.28)	(1.64)	(-1.80)	(-1.77)	(0.22)	(-1.30)
Childless	0.17	-0.38†	-0.23	-0.28	-0.00	0.35
	(0.74)	(-1.65)	(-1.35)	(-1.10)	(-0.01)	(0.78)
Heterosexual	0.06	0.02	0.04	-0.01	0.30	0.47
	(0.38)	(0.14)	(0.34)	(-0.25)	(1.25)	(1.57)
N	977	977	977	977	977	977
*R*^*2*^	0.04	0.03	0.05	0.04	0.02	0.04
Prob. > *F*	0.008	0.344	0.356	0.023	0.000	0.013

*Notes*: Coefficient estimates obtained from ordinary least squares (OLS) regressions. *t*-statistics are given in parentheses, and standard errors are robust to heteroskedasticity.

The references for educational level and religion are college degree and atheism, respectively.

†, ***, **, and * denote significance at the 0.1%, 1%, 5%, and 10% levels, respectively.

## Discussion

Our novel method for quantifying the qualitative responses of a large sample of Australian blood and organ (non-)donors reveals statistically significant differences between blood donors’ and non-donors’ use of the LIWC and MFD word categories in justifying their donation decisions. To the best of the authors’ knowledge, it is the first quantitative study exploring how linguistic features and attitudes predict blood and organ donation behaviour. However, whereas the differences in organ donor versus non-donor use of social and positively emotional terminology are statistically different, such is not the case for their use of egocentric and negatively emotional words. These findings may be explained superficially by the inherent short-term cost differences between blood and organ donation. For example, the negative emotions conjured up by the thought of blood donation (i.e., physical pain) are an easy point of differentiation for those seeking to rationalize or justify a non-donation decision. Conversely, the absence of such negative emotions among organ non-donors may imply that the cognitive processes employed in the organ donation decision do not inform their perceptions of physical pain. Blood donors also incur considerable opportunity costs in their participation (e.g., time, travel, emotional investment, loss of income) that organ donors do not, meaning that the blood non-donors’ use of more egocentric terminology may reflect their recognition and understanding of the individual costs they would incur as a blood donor. If so, targeting non-donors with advertising and marketing communications based on altruistic terminology may not be merely inefficient but totally ineffective as an incentive for behavioural change. Non-donors are clearly already cognisant of the personal costs, and thus no extra amount of pro-social advertising is likely to incentivise a change in behaviour. Similarly, organ donation campaigns that point to no individual cost or negative emotional effect in registering may also fail to increase individual willingness to volunteer as an organ donor given that these factors do not apparently differentiate organ donors from non-donors. Our findings also provide fodder for the broader scientific debate on donor’s motivations being driven by altruism vs. self-reinforcing or obligatory pro-social norms (i.e., doing the right thing). Certainly, statistically significant findings that blood donors (compared to non-donors) use less egocentric (I) words, but also use more morality-based (general and caring) terminology raises questions about the use of “altruism” as the definitive motivation for donors. That the use of caring and fairness moral terms differs between organ donors and non-donors further accentuates this issue. For example, donors may not place as higher priority on fairness rather than emphasizing caring compared with non-donors, who may expect greater reciprocity or see donation as a higher personal cost.

The authors acknowledge the current study has limitations. First, as with most behavioural online surveys our respondents self-selected into the study leading to a convenience sample. That said, this is both unavoidable and necessary in relation to donation research that seeks to compare donor and non-donor populations and behaviour, as donors by their very nature are already a self-selected group. Further, asking individuals about their decision-making regarding being or not being a donor also raises issues of ex-post rationalization, particularly by those who are already donors. That is, donors may simply not be cognizant of the psychology involved in their donation decisions, making the generic terminology used in the donation marketplace (and also as part of this study) their simplest and easiest default justification. The present study did not distinguish blood donor’s responses by their donation frequency, doing so may provide further insights on why someone becomes a frequent donor and should be considered by future studies. That said, the internet is recognised as a valid and valuable tool for medical and health surveys such as this one[35].

Exploring key differences in the terminology employed by individuals to both rationalize and make decisions in medical donation markets is fundamental to increasing both blood and organ donor numbers in the future. Our results suggest that using egocentric, moral or negative emotional terminology when communicating to potential blood and organ donors is (in part) relevant in the blood donation market but unlikely to incentivize or change organ donation behaviour. Further research is thus warranted into how donors and non-donors process and understand the terminology used in this setting and how particular language and communications change or encourage donation behaviour.

## Supporting information

S1 Table Logistic regression–predicting blood donation. *Notes*: Odds ratios obtained from logistic regressions. *z*-statistics are given in parentheses, and standard errors are robust to heteroskedasticity. The references for educational level and religion are college degree and atheism, respectively. †, ***, **, and * denote significance at the 0.1%, 1%, 5%, and 10% levels, respectively.
(DOCX)

S2 Table Logistic regression–predicting organ donation. *Notes*: Odds ratios obtained from logistic regressions. *z*-statistics are given in parentheses, and standard errors are robust to heteroskedasticity. The references for educational level and religion are college degree and atheism, respectively. †, ***, **, and * denote significance at the 0.1%, 1%, 5%, and 10% levels, respectively.
(DOCX)

S3 Table Multinomial logistic regression–predicting blood and organ donation. *Notes*: Relative-risk ratios obtained from multinominal logistic regressions with non-blood and non-organ donor as base outcome. *z*-statistics are given in parentheses, and standard errors are robust to heteroskedasticity. The references for educational level and religion are college degree and atheism, respectively. †, ***, **, and * denote significance at the 0.1%, 1%, 5%, and 10% levels, respectively.
(DOCX)

S4 Table Comparison between blood donation responses by organ donor and non-donor. (DOCX)

S5 Table Comparison between organ donation responses by blood donor and non-donor. (DOCX)
