# Supplementary material for: Exploring the impact of terminology differences in blood and organ donor decision making
Source: PLoS One. 2020 Jan 9;15(1):e0227536. doi: 10.1371/journal.pone.0227536 (PMC6952186; doi:10.1371/journal.pone.0227536)
Supplement: S1 Table — Notes: Odds ratios obtained from logistic regressions. z-statistics are given in parentheses, and standard errors are robust to heteroskedasticity. The references for educational level and religion are college degree and atheism, respectively. †, ***, **, and * denote significance at the 0.1%, 1%, 5%, and 10% levels, respectively. (DOCX) [file pone.0227536.s001.docx]

# Supporting information

**S1 Table. Logistic regression – predicting blood donation**

| *Dep. Var.: Blood donation* | All | | Female | | Male | |
| --- | --- | --- | --- | --- | --- | --- |
| *Indep. Var.* | (1) | | (2) | | (3) | |
| Male | 0.94 | (-0.30) |  |  |  |  |
| Age | 1.02* | (2.08) | 1.01 | (1.13) | 1.07** | (3.21) |
| Height (cm) | 1.33 | (1.40) | 1.08 | (0.21) | 0.91 | (-0.20) |
| Height^2^ | 1.00 | (-1.42) | 1.00 | (-0.23) | 1.00 | (0.28) |
| Weight (kg) | 1.04 | (1.20) | 1.11* | (2.03) | 1.14† | (1.65) |
| Weight^2^ | 1.00 | (-1.09) | 1.00† | (-1.92) | 1.00 | (-1.51) |
| *ln*(Income) | 1.46** | (3.11) | 1.63*** | (3.34) | 1.41 | (1.28) |
| *Education* |  |  |  |  |  |  |
| High School | 0.78 | (-1.41) | 0.78 | (-1.15) | 1.21 | (0.50) |
| Post-Graduate | 1.64** | (2.65) | 1.76* | (2.40) | 0.54 | (-1.35) |
| Single | 1.25 | (1.24) | 1.54* | (2.00) | 1.93 | (1.41) |
| *Religion* |  |  |  |  |  |  |
| Buddhism | 0.82 | (-0.63) | 0.84 | (-0.49) | 1.00 | (.) |
| Christianity | 0.69* | (-2.12) | 0.77 | (-1.23) | 0.49* | (-1.96) |
| Hinduism | 1.65 | (1.27) | 1.16 | (0.21) | 0.17 | (-1.62) |
| Islam | 1.02 | (0.05) | 0.67 | (-0.67) | 0.21* | (-2.02) |
| Judaism | 0.61 | (-0.36) | 0.70 | (-0.26) |  |  |
| Other | 0.63* | (-2.15) | 0.63† | (-1.81) | 0.18** | (-3.25) |
| Childless | 0.99 | (-0.06) | 0.90 | (-0.36) | 0.41† | (-1.78) |
| Heterosexual | 0.97 | (-0.15) | 0.84 | (-0.77) | 1.16 | (0.29) |
| N | 977 |  | 644 |  | 319 |  |
| Pseudo *R^2^* | 0.08 |  | 0.08 |  | 0.22 |  |
| Prob. > *χ^2^* | 0.000 |  | 0.000 |  | 0.000 |  |

*Notes*: Odds ratios obtained from logistic regressions. *z*-statistics are given in parentheses, and standard errors are robust to heteroskedasticity.

The references for educational level and religion are college degree and atheism, respectively.

†, ***, **, and * denote significance at the 0.1%, 1%, 5%, and 10% levels, respectively.
