# Supplementary material for: Exploring the impact of terminology differences in blood and organ donor decision making
Source: PLoS One. 2020 Jan 9;15(1):e0227536. doi: 10.1371/journal.pone.0227536 (PMC6952186; doi:10.1371/journal.pone.0227536)
Supplement: S2 Table — Notes: Odds ratios obtained from logistic regressions. z-statistics are given in parentheses, and standard errors are robust to heteroskedasticity. The references for educational level and religion are college degree and atheism, respectively. †, ***, **, and * denote significance at the 0.1%, 1%, 5%, and 10% levels, respectively. (DOCX) [file pone.0227536.s002.docx]

**S2 Table. Logistic regression – predicting organ donation**

| *Dep. Var.: Organ donation* | All | | Female | | Male | |
| --- | --- | --- | --- | --- | --- | --- |
| *Indep. Var.* | (1) | | (2) | | (3) | |
| Male | 0.22*** | (-6.76) |  |  |  |  |
| Age | 1.05*** | (4.20) | 1.04** | (3.00) | 1.07** | (3.21) |
| Height (cm) | 0.79 | (-1.14) | 1.11 | (0.25) | 0.91 | (-0.20) |
| Height^2^ | 1.00 | (1.24) | 1.00 | (-0.23) | 1.00 | (0.28) |
| Weight (kg) | 1.04 | (1.26) | 1.04 | (0.91) | 1.14† | (1.65) |
| Weight^2^ | 1.00 | (-0.93) | 1.00 | (-0.61) | 1.00 | (-1.51) |
| *ln*(Income) | 1.50** | (2.79) | 1.57* | (2.54) | 1.41 | (1.28) |
| *Education* |  |  |  |  |  |  |
| High School | 1.01 | (0.07) | 0.97 | (-0.16) | 1.21 | (0.50) |
| Post-Graduate | 0.69† | (-1.74) | 0.72 | (-1.29) | 0.54 | (-1.35) |
| Single | 1.06 | (0.32) | 0.92 | (-0.38) | 1.93 | (1.41) |
| *Religion* |  |  |  |  |  |  |
| Buddhism | 0.23*** | (-3.69) | 0.29** | (-2.87) | 1.00 | (.) |
| Christianity | 0.58** | (-3.06) | 0.62* | (-2.23) | 0.49* | (-1.96) |
| Hinduism | 0.12** | (-2.64) | 0.10* | (-2.08) | 0.17 | (-1.62) |
| Islam | 0.22** | (-2.89) | 0.25† | (-1.88) | 0.21* | (-2.02) |
| Judaism | 2.56 | (0.82) | 2.43 | (0.77) |  |  |
| Other | 0.33*** | (-4.64) | 0.39*** | (-3.49) | 0.18** | (-3.25) |
| Childless | 0.54* | (-2.39) | 0.57† | (-1.81) | 0.41† | (-1.78) |
| Heterosexual | 1.74** | (2.64) | 1.79* | (2.55) | 1.16 | (0.29) |
| N | 977 |  | 644 |  | 319 |  |
| Pseudo *R^2^* | 0.20 |  | 0.16 |  | 0.22 |  |
| Prob. > *χ^2^* | 0.000 |  | 0.000 |  | 0.000 |  |

*Notes*: Odds ratios obtained from logistic regressions. *z*-statistics are given in parentheses, and standard errors are robust to heteroskedasticity.

The references for educational level and religion are college degree and atheism, respectively.

†, ***, **, and * denote significance at the 0.1%, 1%, 5%, and 10% levels, respectively.
