# Supplementary material for: Exploring the impact of terminology differences in blood and organ donor decision making
Source: PLoS One. 2020 Jan 9;15(1):e0227536. doi: 10.1371/journal.pone.0227536 (PMC6952186; doi:10.1371/journal.pone.0227536)
Supplement: S3 Table — Notes: Relative-risk ratios obtained from multinominal logistic regressions with non-blood and non-organ donor as base outcome. z-statistics are given in parentheses, and standard errors are robust to heteroskedasticity. The references for educational level and religion are college degree and atheism, respectively. †, ***, **, and * denote significance at the 0.1%, 1%, 5%, and 10% levels, respectively. (DOCX) [file pone.0227536.s003.docx]

**S3 Table. Multinomial logistic regression – predicting blood and organ donation**

| *Outcome* | Blood-only donor | | Organ-only donor | | Blood and organ donor | |
| --- | --- | --- | --- | --- | --- | --- |
| *Indep. Var.* |  | |  | |  | |
| Male | 1.14 | (0.55) | 0.21*** | (-5.50) | 0.25*** | (-4.55) |
| Age | 1.03† | (1.86) | 1.05** | (3.26) | 1.06*** | (3.98) |
| Height (cm) | 1.70† | (1.73) | 0.84 | (-0.75) | 0.97 | (-0.11) |
| Height^2^ | 1.00† | (-1.80) | 1.00 | (0.79) | 1.00 | (0.18) |
| Weight (kg) | 1.08 | (1.46) | 1.07 | (1.64) | 1.06 | (1.35) |
| Weight^2^ | 1.00 | (-1.35) | 1.00 | (-1.37) | 1.00 | (-1.09) |
| *ln*(Income) | 1.25 | (1.13) | 1.31 | (1.47) | 1.92*** | (3.61) |
| *Education* | ref. |  | ref. |  | ref. |  |
| High School | 1.08 | (0.32) | 1.34 | (1.29) | 0.67 | (-1.48) |
| Post-Graduate | 1.66* | (2.03) | 0.63 | (-1.64) | 1.02 | (0.08) |
| Single | 0.96 | (-0.19) | 0.77 | (-1.13) | 1.48 | (1.47) |
| *Religion* |  |  |  |  |  |  |
| Buddhism | 1.25 | (0.60) | 0.31* | (-2.32) | 0.19** | (-2.99) |
| Christianity | 0.75 | (-1.18) | 0.61* | (-2.17) | 0.45*** | (-3.34) |
| Hinduism | 2.53* | (2.15) | 0.21 | (-1.46) | 0.15† | (-1.71) |
| Islam | 1.38 | (0.84) | 0.29† | (-1.75) | 0.23* | (-2.07) |
| Other | 1.09 | (0.33) | 0.56* | (-2.17) | 0.18*** | (-4.80) |
| Childless | 1.41 | (1.00) | 0.67 | (-1.12) | 0.53† | (-1.92) |
| Heterosexual | 0.89 | (-0.52) | 1.66* | (1.99) | 1.75† | (1.89) |
| N | 977 |  |  |  |  |  |
| Pseudo *R^2^* | 0.14 |  |  |  |  |  |
| Prob. > *χ^2^* | 0.000 |  |  |  |  |  |

*Notes*: Relative-risk ratios obtained from multinominal logistic regressions with non-blood and non-organ donor as base outcome. *z*-statistics are given in parentheses, and standard errors are robust to heteroskedasticity.

The references for educational level and religion are college degree and atheism, respectively.

†, ***, **, and * denote significance at the 0.1%, 1%, 5%, and 10% levels, respectively.
