# Supplementary material for: Exploring the impact of terminology differences in blood and organ donor decision making
Source: PLoS One. 2020 Jan 9;15(1):e0227536. doi: 10.1371/journal.pone.0227536 (PMC6952186; doi:10.1371/journal.pone.0227536)
Supplement: S4 Table — (DOCX) [file pone.0227536.s004.docx]

**S4 Table.** **Comparison between blood donation responses by organ donor and non-donor.**

| Category | Blood donation response | Type | |  |  |
| --- | --- | --- | --- | --- | --- |
|  |  | Donor (organ) | Non-donor (organ) | *t* | *p* |
| *i* | Donor | 7.07 | 7.57 | 0.756 | 0.450 |
|  | Non-donor | 9.22 | 10.73 | 2.450 | 0.015 |
| *social* | Donor | 8.60 | 8.28 | -0.332 | 0.740 |
|  | Non-donor | 1.28 | 1.58 | 1.113 | 0.266 |
| *posemo* | Donor | 3.43 | 3.85 | 0.751 | 0.453 |
|  | Non-donor | 1.36 | 1.41 | 0.188 | 0.851 |
| *negemo* | Donor | 0.37 | 0.56 | 1.127 | 0.260 |
|  | Non-donor | 3.64 | 3.79 | 0.190 | 0.849 |
| *moral* | Donor | 0.96 | 1.11 | 0.469 | 0.639 |
|  | Non-donor | 0.24 | 0.27 | 0.203 | 0.839 |
| *care* | Donor | 0.34 | 0.42 | 0.355 | 0.723 |
|  | Non-donor | 0.00 | 0.08 | 1.542 | 0.123 |
| *fairness* | Donor | 0.83 | 1.04 | 0.622 | 0.534 |
|  | Non-donor | 1.09 | 1.14 | 0.192 | 0.848 |

Mean comparison *t-*test. N of organ and blood donor = 191; N of blood only donor = 188; N of organ and blood non-donor = 458; N of organ only donor =198. Two-tailed *P*-values.
