# Supplementary material for: Exploring the impact of terminology differences in blood and organ donor decision making
Source: PLoS One. 2020 Jan 9;15(1):e0227536. doi: 10.1371/journal.pone.0227536 (PMC6952186; doi:10.1371/journal.pone.0227536)
Supplement: S5 Table — (DOCX) [file pone.0227536.s005.docx]

**S5 Table.** **Comparison between organ donation responses by blood donor and non-donor.**

| Category | Organ donation response | Type | |  |  |
| --- | --- | --- | --- | --- | --- |
|  |  | Donor (blood) | Non-donor (blood) | *t* | *p* |
| *i* | Donor | 10.74 | 10.30 | -0.643 | 0.521 |
|  | Non-donor | 11.09 | 10.93 | -0.244 | 0.807 |
| *social* | Donor | 7.33 | 7.49 | 0.195 | 0.846 |
|  | Non-donor | 1.87 | 1.83 | -0.111 | 0.912 |
| *posemo* | Donor | 4.32 | 4.54 | 0.370 | 0.712 |
|  | Non-donor | 2.57 | 2.11 | -1.052 | 0.293 |
| *negemo* | Donor | 0.30 | 0.44 | 0.952 | 0.342 |
|  | Non-donor | 1.33 | 0.54 | -1.734 | 0.083 |
| *moral* | Donor | 0.48 | 0.60 | 0.471 | 0.638 |
|  | Non-donor | 0.19 | 0.27 | 0.544 | 0.587 |
| *care* | Donor | 0.57 | 0.53 | -0.149 | 0.882 |
|  | Non-donor | 0.01 | 0.03 | 0.561 | 0.575 |
| *fairness* | Donor | 0.77 | 0.85 | 0.338 | 0.736 |
|  | Non-donor | 2.05 | 1.45 | -1.348 | 0.178 |

Mean comparison *t-*test. N of organ and blood donor = 191; N of blood only donor = 188; N of organ and blood non-donor = 458; N of organ only donor =198. Two-tailed *P*-values.
